# PSYCHOSOCIAL AND IMMIGRATION DETERMINANTS OF SLEEP HEALTH AMONG OLDER CHINESE IMMIGRANTS

**DOI:** 10.1093/geroni/igae098.0376

**Published:** 2024-12-31

**Authors:** Yanping Jiang, Fengyan Tang

**Affiliations:** Rutgers, The State University of New Jersey, New Brunswick, New Jersey, United States; University of Pittsburgh, Wexford, Pennsylvania, United States

## Abstract

Good sleep is critical for maintaining physical and cognitive health among older adults. Few studies, however, have examined factors related to sleep among older Asian immigrants, who face unique challenges that may put them at elevated risk of experiencing poor sleep. This study aimed to examine the effect of various psychosocial and immigrant factors on sleep outcomes over 2 years in a large sample of older Chinese American immigrants. Data were drawn from a subsample (N = 2,228) from the Population Study of Chinese Elderly in Chicago. Participants self-reported multiple psychosocial and immigrant factors, including neighborhood cohesion, neighborhood disorder, social disconnectedness, loneliness, perceived racial discrimination, perceived social support, negative social interactions, acculturation, perceived stress, depression, length of living in the U.S., income, and education. Participants also completed questionnaires on sleep quality and insomnia. Multivariate regression analyses showed that of the psychosocial and immigrant factors, depressive symptoms significantly predicted poorer sleep quality and worse insomnia over 2 years, controlling for baseline sleep quality and insomnia. Also, neighborhood disorder predicted worse insomnia over 2 years but not sleep quality. In addition, exploratory analyses for subdomains of sleep quality showed that perceived stress and negative social interactions predicted lower sleep efficiency over time. Neighborhood disorder and depressive symptoms appear to be salient risk factors for poor sleep health among older Chinese immigrants. Interventions targeting neighborhood environment and mental health services may be critical in improving sleep health and promoting healthy aging of older Chinese immigrants.

